# Confined Electrochemiluminescence Generation at Ultra-High-Density Gold Microwell Electrodes

**DOI:** 10.3389/fchem.2020.630246

**Published:** 2021-01-26

**Authors:** Jialian Ding, Ping Zhou, Weiliang Guo, Bin Su

**Affiliations:** Department of Chemistry, Institute of Analytical Chemistry, Zhejiang University, Hangzhou, China

**Keywords:** electrochemiluminescence, microwell electrode array, imaging, confinement, reaction mechanisms

## Abstract

Electrochemiluminescence (ECL) imaging analysis based on the ultra-high-density microwell electrode array (UMEA) has been successfully used in biosensing and diagnostics, while the studies of ECL generation mechanisms with spatial resolution remain scarce. Herein we fabricate a gold-coated polydimethylsiloxane (PDMS) UMEA using electroless deposition method for the visualization of ECL reaction process at the single microwell level in conjunction with using microscopic ECL imaging technique, demonstrating that the microwell gold walls are indeed capable of enhancing the ECL generation. For the classical ECL system involving tris(2,2′-bipyridyl)ruthenium (Ru(bpy)_3_^2+^) and tri-*n*-propylamine (TPrA), the ECL image of a single microwell appears as a surface-confined ring, indicating the ECL intensity generated inside the well is much stronger than that on the top surface of UMEA. Moreover, at a low concentration of Ru(bpy)_3_^2+^, the ECL image remains to be ring-shaped with the increase of exposure time, because of the limited lifetime of TPrA radical cations TPrA^+•^. In combination with the theoretical simulation, the ring-shaped ECL image is resolved to originate from the superposition effect of the mass diffusion fields at both microwell wall and bottom surfaces.

## Introduction

Electrochemiluminescence (ECL) is light emission from excited state luminophores generated by electrochemical reactions, which possesses better spatiotemporal control in comparison with chemiluminescence (Richter, 2004; Hu and Xu, 2010; Hesari and Ding, 2017; Liu et al., 2019). Because no external light illumination is required to generate ECL emission, ECL has unique advantages, such as low background and high sensitivity, et al. It has manifested itself to be a powerful transduction signal for the determination of small biomolecules, DNA and protein biomarkers in biosensing and clinical diagnostics (Doeven et al., 2014; Wu et al., 2014; Lim et al., 2016; Bist et al., 2017; Ji et al., 2017; Yang et al., 2018; Zhou et al., 2018; Zhang et al., 2019a; Jones et al., 2020). Moreover, ECL has recently emerged as a useful surface analysis technology because of its intrinsic surface-confined and surface-sensitive nature (Guo et al., 2017; Zhang et al., 2019b; Zanut et al., 2019; Ma et al., 2020). Compared with the conventional intensity-based ECL measurement of the entire electrochemical ensembles, ECL imaging can function as a diagnostic tool to elucidate the relationship between the topography/structures and electron transfer properties of electrode surface with spatial resolution (Pantano and Kuhr, 1993; Hopper and Kuhr, 1994). Therefore, it has been successfully employed to visualize latent fingerprints, micro-/nanoparticles, cells and subcellular structures at the single entity level (Xu et al., 2012; Xu et al., 2014; Valenti et al., 2017; Ma et al., 2018; Voci et al., 2018; Zhu et al., 2018; Ding et al., 2020; Zhang et al., 2020). In addition, ECL imaging also provides an addressable approach to map the spatial distribution of ECL generation on a single bead surface, proving the existence of low oxidation potential route for ECL generation (Sentic et al., 2014; Dutta et al., 2020).

Microporous electrode array consisting of physically separated electrodes on a substrate surface allows optically addressable and high-throughput analysis using ECL imaging technique (Wu et al., 2015; Xu et al., 2017; Xia et al., 2018). Compared with the plate electrode, microporous electrode array has a high surface/volume ratio and interconnectivity of individual micrometer-pore electrodes. Thus, the signals generated by individual electrodes in the whole array can be all read at once and spatially resolved, allowing the simultaneous detection of multiple targets in a single workflow (Deiss et al., 2009; Zhang et al., 2014; Cui et al., 2020). Previous studies have mainly focused on the application of microelectrode arrays for the quantitative determination of biomolecules, such as glucose, nucleic acid, proteins and cells, suggesting the porous electrode array is very useful in biosensing (Deiss et al., 2009; Liu et al., 2016; Xu et al., 2016; Xia et al., 2018). While those concerning the ECL reaction mechanisms are scarce (Chovin et al., 2004). Very recently, we have reported the measurement of the thickness of ECL-emitting layer using microtube electrode ensembles, showing that the combined use of microelectrode ensembles and ECL imaging technique is indeed a powerful tool for deciphering ECL reaction mechanisms (Guo et al., 2020).

**SCHEME 1:**
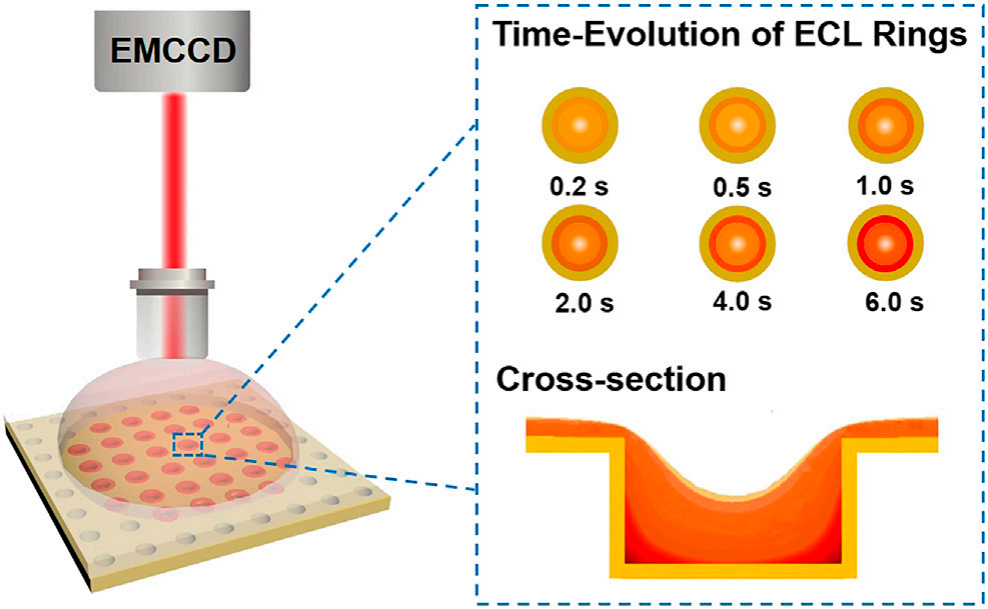
Illustration of confined ECL generation at the microwell electrode array probed by ECL microscopy. EMCCD = electron multiplying charge coupled device.

Here we fabricate an ultra-high-density of polydimethylsiloxane (PDMS) based microwell electrode array (UMEA) by micro-/nanofabrication and electroless deposition of gold (LaFratta and Walt, 2008; Cui et al., 2020). As shown in Scheme 1, the whole surface of microwell array is continuously coated with an ultrathin layer of gold, meaning that its top/bottom surface and vertical sidewall is all capable of triggering ECL reactions. While the brightest ECL emission is generated around the orifices of microwells and confined inside each microwells, as revealed by ECL microscopy, generating an ensemble of ECL emitting rings. In combination with digital simulations, the ring-shaped ECL emission is resolved to originate from the superposition of radial and axial mass diffusion fields inside microwells. At a low concentration of tris(2,2′-bipyridyl)ruthenium (Ru(bpy)_3_^2+^), the ECL image of a single microwell remains to be ring-shaped and independent on the exposure time of CCD camera, which can be ascribed to the limited lifetime of tri-*n*-propylamine (TPrA)-derived radicals. This work proves the distribution of ECL emission can be modulated in a confined volume. The vertical walls of gold microwells favor the mass transport of coreactant radicals from the electrode surface to beads, enhancing the ECL generation from the so-called low oxidation potential route, thus promoting the bead-based ECL detection sensitivity when loading single beads onto single microwells. We believe this confinement and enhancement effect can be useful for promoting the ECL detection sensitivity based on the micrometer pore electrode.

## Materials and Methods

### Chemicals and Reagents

All chemicals of analytical grade or higher were used as received without further purification. Ultrapure water (18.2 MΩ⋅cm) was employed to prepare all aqueous solutions. SU-8 mold on silicon wafer was fabricated by Suzhou Yancai Micro Nano Technology Co., Ltd. Sylgard 184 polydimethylsiloxane (PDMS) was purchased from Dow Corning. Conductive copper tape and gold film electrode were bought from Hyde Venture (Beijing) Biotechnology Co., Ltd. and Dynasil’s Companies, respectively. Sodium gold sulfite (Na_3_Au(SO_3_)_2_, 0.25 M) was bought from Changzhou Institute of Chemistry Research. Methanol (CH_3_OH, 99.0%), formaldehyde (HCHO, 37–40%), sodium sulfite (Na_2_SO_3_), sodium dihydrogen phosphate dihydrate (NaH_2_PO_4_·2H_2_O, 99.0%), disodium hydrogen phosphate dodecahydrate (Na_2_HPO_4_·12H_2_O, 99.0%), potassium nitrate (KNO_3_, 99.0%) and concentrated sulfuric acid (H_2_SO_4_, 95%–98%) were bought from Sinopharm Chemical Reagent. Trifluoroacetate (CF_3_COOH, 99.5%), silver nitrate (AgNO_3_, 99.8%), potassium ferricyanide (K_3_Fe(CN)_6_, 99.0%) and potassium ferrocyanide (K_4_Fe(CN)_6_, 99.0%) and tri-*n*-propylamine (TPrA, 99%) were purchased from Aladdin. Stannous chloride (SnCl_2_, 97.5%) was bought from J&K Scientific Ltd. Tris(2,2′-bipyridyl)ruthenium dichloride hexahydrate (Ru(bpy)_3_Cl_2_·6H_2_O, 99.95%) and ammonium hydroxide solution (NH_3_·H_2_O, 25 wt.%) were obtained from Sigma-Aldrich. Phosphate buffer (PB, 0.1 M, pH 7.4) served as the supporting electrolyte for electrochemical measurement.

### Preparation and Characterization of Gold-Coated UMEA 

**FIGURE 1:**
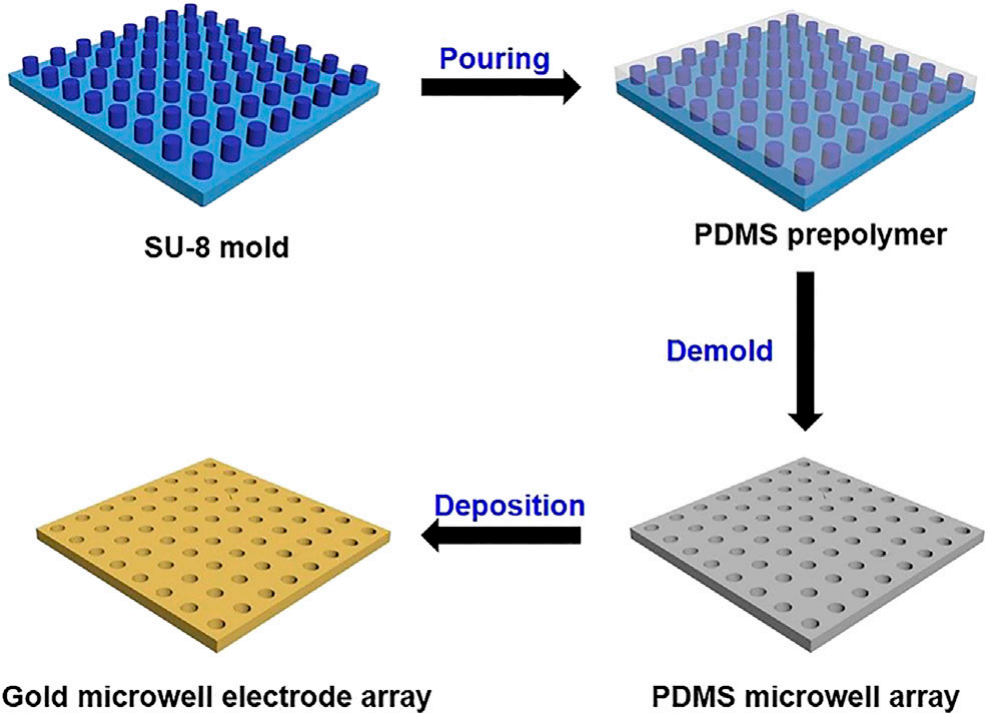
Schematic procedure for fabrication of gold-coated UMEA.

UMEA was prepared using SU-8 micropillar array and electroless deposition of gold (see the fabrication process shown in Figure 1). The micropillars possess uniform cylinder structures with ordered alignments and high density (1.15 × 10^6^/cm^2^). The diameter and height of each cylinder are 5 and 2.5 µm, respectively. Briefly, the PDMS monomer and curing agent (w/w, 10/1) were uniformly mixed, cooled in refrigerator at 4°C for 30 min, and then poured onto the silica wafer mold with an array of SU-8 micropillars. Subsequently, the wafer was baked on a hot plate at 85°C for 30 min. Finally, the cured PDMS was stripped from the mold to obtain the microwell array (ca. 5 μm in diameter, ca. 2.5 μm in depth). The gold microwell electrode was prepared using the electroless gold plating (Pereira et al., 2006; Bottari et al., 2014). Typically, the PDMS substrate was sequentially immersed in methanol for 2 h, methanol/water (v/v, 1/1) containing 0.026 M SnCl_2_ and 0.07 M trifluoroacetic acid for 45 min. After rinsing with methanol, the PMDS was immersed in a freshly prepared solution of 0.029 M [Ag (NH_3_)_2_]NO_3_ for 10 min. After rinsing again with methanol and water, it was immersed into the gold plating bath containing 8.0 × 10^–3^ M Na_3_Au(SO_3_)_2_, 0.127 M Na_2_SO_3_ and 0.625 M formaldehyde at 4°C. After being plated for 6 h, the whole surface of PDMS was coated with a thin layer of gold. The gold-coated PDMS was first placed on a glass substrate, and one corner of the deposited gold layer was then put in contact with the conductive copper tape for connecting the working electrode and electrochemical workstation. Finally, the electrode was rinsed with ultrapure water and dried with nitrogen stream for use.

Field emission scanning electron microscope (SEM, Hitachi, SU8010) was employed to characterize the morphology of microwell electrode array. The operating voltage of SEM testing was 3 kV. Electrochemical measurements were carried out on an electrochemistry workstation (CHI440, Shanghai, China). All measurements were conducted using the classic three-electrode configuration, in which the gold microwell electrode array, a platinum wire and a silver/silver chloride electrode functioned as the working, counter and reference electrodes, respectively.

### Electrochemiluminescence Imaging Measurement

All ECL measurements were conducted using the classic three-electrode configuration as described above. The synchronous acquisition of the ECL intensity-voltage curves overlaid with cyclic voltammetry curves (CVs) was performed on an MPI-E ECL analytical system (Remex Analysis Instrument, Xi’an, China). The PMT was biased at 200 V, and the scan rate was 0.1 V/s. ECL images at different potentials were obtained with a custom microscopic ECL imaging system, consisting of electron multiplying charge coupled device (EMCCD) camera, water immersion objective (Nikon, CFI Apo 40×, N.A. 0.8) and CHI 832C electrochemical workstation (CHI Instrument, Shanghai, China). In each imaging experiment, the double-step potential was applied at the gold microwell electrode array, while the EMCCD exposure time was in consistent with the time of double-step potential.

## Results and Discussion

### Characterization of Gold-Coated UMEA

**FIGURE 2:**
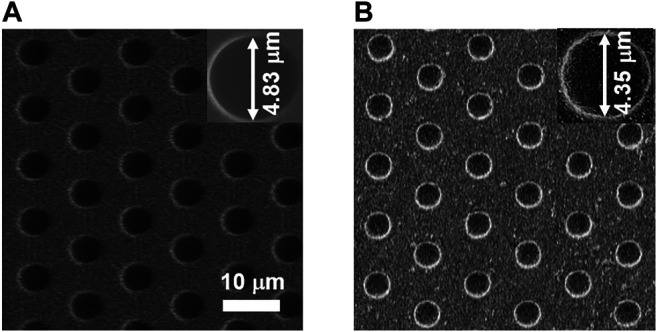
SEM images of PDMS microwell array **(A)** and gold-coated UMEA **(B)**. The scale bar is 10 µm. The insets show the magnified SEM images of single microwells.

The morphology and structure of bare PDMS microwell array and gold-coated UMEA were first characterized by SEM measurement. From the top view of SEM images shown in Figures 2A,B both PDMS and UMEA arrays consist of a highly uniform distribution of microwells over a large area, and the circular wells are hexagonally aligned with 10 µm center-to-center spacing. The well diameters of two arrays are 4.83 and 4.35 µm, respectively, and the gold wall thickness of UMEA is therefore ca. 200 nm. The depth of microwells was estimated to be ca. 2.5 µm according to the height of SU-8 micropillars.

**FIGURE 3:**
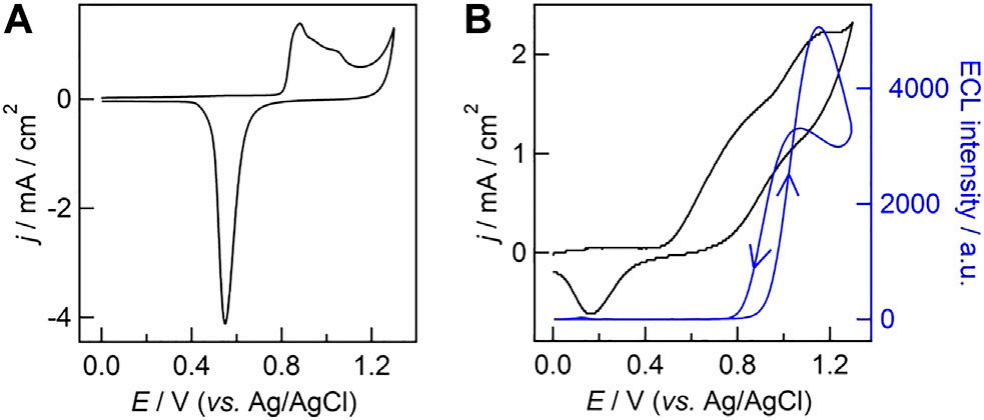
**(A)** Cyclic voltammogram (CV) obtained with gold-coated UMEA in 0.1 M H_2_SO_4_. **(B)** ECL intensity−potential curve (blue line) overlaid with CV (black line) obtained with gold-coated UMEA in phosphate buffer (PB, 0.1 M, pH 7.4) containing 500 μM Ru(bpy)_3_^2+^ and 25 mM TPrA. The scan rate was 0.1 V/s. The photomultiplier tube was biased at 200 V.

Cyclic voltammetry was also performed to study the electrochemical performance of UMEA in 0.1 M H_2_SO_4_. As shown in Figure 3A, the characteristic redox peaks of gold electrode were observed with gold-coated UMEA, including one reduction current peak at +0.56 V (vs. Ag/AgCl, the same in the following) and three successive oxidation ones in the potential range from +0.88 V to +1.05 V. We then measured ECL behavior of the classic co-reactant ECL system involving Ru(bpy)_3_^2+^ and TPrA at UMEA, and the ECL intensity-potential curve (blue line, the arrows indicate the directions of scanned potential) overlaid with CV (black line) were presented in Figure 3B. A significant anodic current wave was clearly observed when the oxidation potential scanned from 0 V to +0.5 V, which can be ascribed to the overlapped oxidation of gold electrode itself and TPrA in PB solution. Once the applied potential was up to ca. +0.9 V (namely the redox potential of Ru(bpy)_3_^2+^, see Supplementary Figure S1), the ECL generation was launched, and the ECL intensity increased with the increase of potential and reached the maximum at +1.1 V. When scanning the potential backward from +1.3 V, the ECL intensity reached another maximum value at ca. +1.0 V.

### ECL Imaging of Gold-Coated UMEA 

**FIGURE 4:**
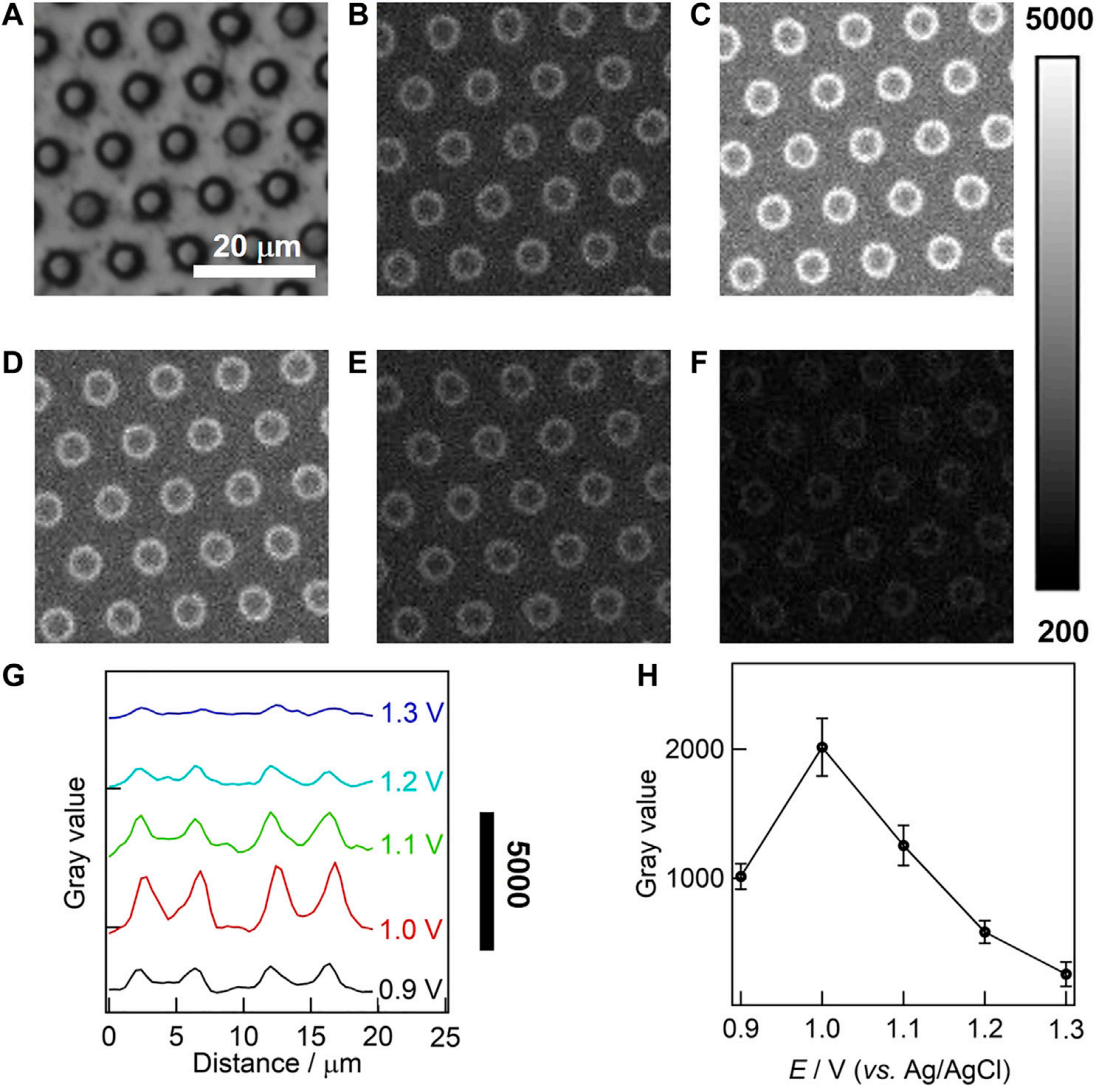
Bright field (BF, **A)** and ECL **(B–F)** images of gold-coated UMEA in PB (0.1 M, pH 7.4) containing 50 μM Ru(bpy)_3_^2+^ and 50 mM TPrA. The ECL imaging were triggered by a double-step potential (initial potential 0 V; respective pulse potentials 0.9, 1.0, 1.1, 1.2, 1.3 V; pulse period 2 s; pulse time 1 s). The exposure time of EMCCD was 4 s. **(G)** The grayscale variation of ECL along the radial direction of two adjacent microwells. **(H)** The variation of ECL intensity with the potential at the gold-coated UMEA at single microwell levels.

The effect of oxidation potential on ECL intensity was studied at the single microwell level by microscopy, and a double-step potential was applied to trigger the ECL generation from 50 µM Ru(bpy)_3_^2+^ and 50 mM TPrA. In this case, the initial potential was set to 0 V (lower than +0.17 V for the reduction potential of gold electrode, see Supplementary Figure S1), and the pulse potential was set in the range from +0.9 to +1.3 V with a potential interval of 0.1 V. Prior to ECL imaging, the focal plane was adjusted to the orifices of UMEA under the reflected bright-field mode using a halogen lamp. The external light source was then switched off, and the double-step potential was applied for 4 s, which was consistent with the exposure time of EMCCD. The stability of gold-coated UMEA was investigated by the multicycle ECL measurement, showing that the relative standard deviation of ECL intensities extracted from sequential ECL images was only 6% (see Supplementary Figure S2). Figures 4A–F compares BF and ECL images obtained at different pulse potentials for the same region of interest of UMEA. Though the whole surface of UMEA is continuously coated with gold film, the emission array with ring-shaped ECL patterns is clearly observed around the orifices of microwells. The average diameter of ECL rings is 4.12 ± 0.20 μm from the statistical measurement of 100 microwells, which is smaller than the physical size of 4.35 µm estimated from SEM image. That is to say, the ECL generation was restricted and enhanced in the confined geometries, allowing the spatial resolution of ECL rings in the same solution. For more quantitative analysis of light distribution, the grayscale variations of ECL images along the radial direction of two adjacent microwells were plotted in Figure 4G, and the ECL intensity profiles have the similar tendency, showing clearly the increase of light intensity at the sidewall of gold microwells. And the ECL intensities gradually decreased away from the sidewall surface in both directions, while the values obtained at the microwell centers were slightly higher than those obtained from the top surface of UMEA. As shown in Supplementary Figure S3, the ECL intensity obtained at the gold-coated UMEA was at least seven times higher than that of the commercial gold film electrode. Furthermore, the ECL intensity at the single microwell level varies with the potential, as displayed in Figure 4H, showing that the pulse potential of 1.0 V is optimal for ECL imaging. In addition, the imaging analysis of gold-coated UMEA was also performed at a high concentration of Ru(bpy)_3_^2+^ (500 µM, see corresponding ECL images in Supplementary Figure S4). The variation of ECL intensity with potential is similar to that at the low luminophore concentration.

**FIGURE 5:**
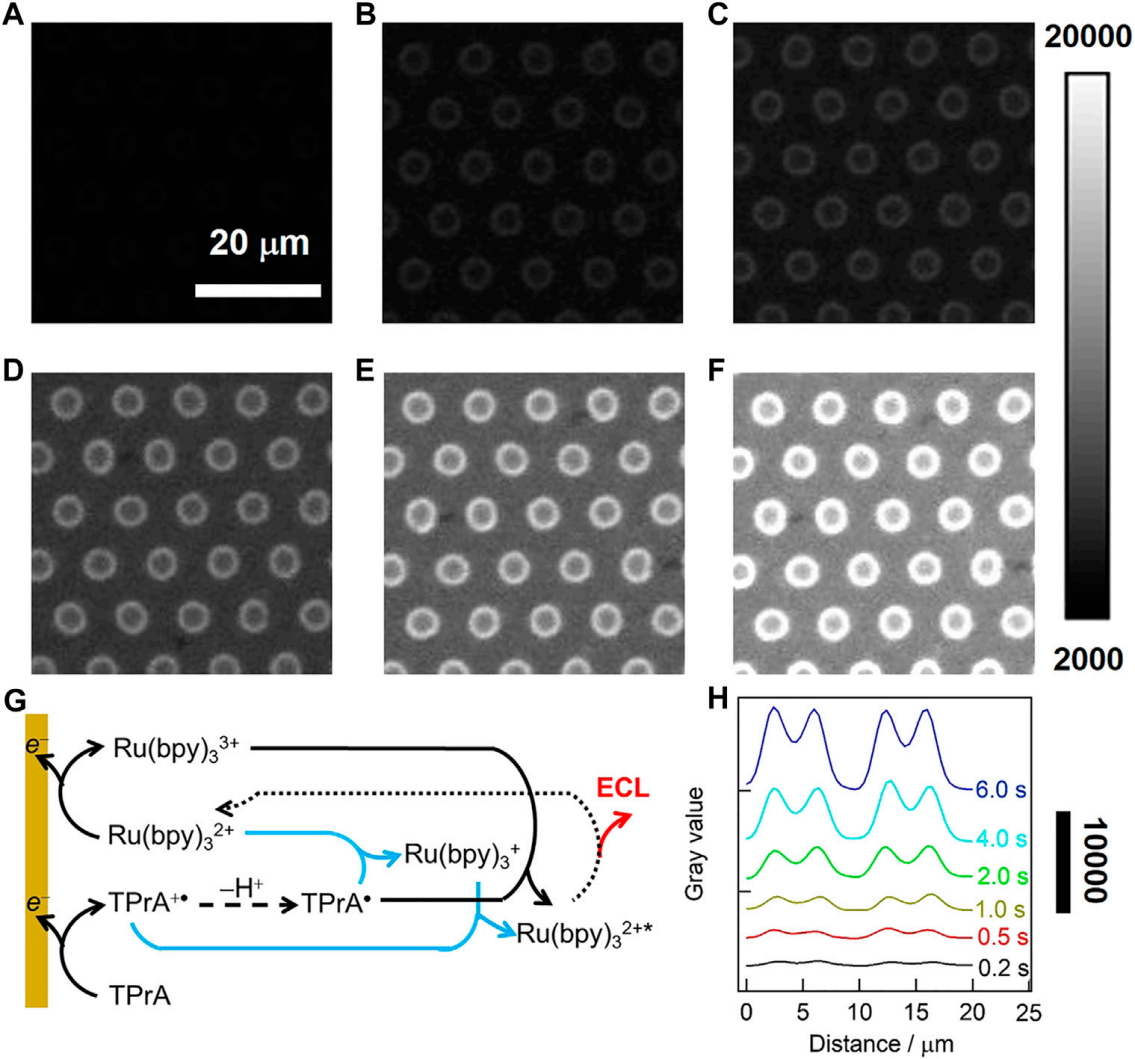
**(A–F)** ECL images in 0.1 M·PB (pH 7.4) containing 50 μM Ru(bpy)_3_^2+^ and 50 mM TPrA. A double-step potential (initial potential 0 V, pulse potential 1.0 V) was applied to launch the ECL reaction. The pulse time was 0.2 s **(A)**, 0.5 s **(B)**, 1 s **(C)**, 2 s **(D)**, 4 s **(E)** and 6 s **(F)**. The exposure time of EMCCD was in consistent with the time of the double-step potential. **(G)** ECL reaction pathways for Ru(bpy)_3_^2+^/TPrA system at a low luminophore concentration. Oxidative reduction route (black line) is concomitant with LOP route (blue lines). **(H)** The grayscale variation of ECL along the radial direction of two adjacent microwells under different exposure time.

We further investigate the evolution of ECL rings with the exposure time of EMCCD (also namely the pulse time), and the potential was pulsed to optimized value of 1.0 V. ECL images obtained with 50 µM Ru(bpy)_3_^2+^/50 mM TPrA under different exposure time were displayed in Figures 5A–F, and it turned brighter with the increase of the time from 0.2 to 6 s, but remained to be ring-shaped. In this case, the ECL generation can be described by the classical oxidative-reduction route (outlined with black lines in Figure 5G). Namely, both Ru(bpy)_3_^2+^ and TPrA are directly oxidized to form Ru(bpy)_3_^3+^ and TPrA^+•^ at the electrode surface. TPrA^+•^ is then deprotonated to generate strongly reductive radical TPrA^•^, which further reacts with Ru(bpy)_3_^3+^ via homogeneous electron transfer reaction for producing the excited state Ru(bpy)_3_^2+^* that emits ECL light (Miao et al., 2002). And the low oxidation potential route (marked with blue lines in Figure 5G) might occur simultaneously. In both routes, the ECL patterns are essentially determined by the concentration profiles of TPrA^+•^, because of its limited lifetime of 0.2 ms (Miao et al., 2002). As shown in Figure 5H, the grayscale variations of ECL rings have the similar tendency under different exposure time, suggesting the ECL rings remain unchanged and are independent on the exposure time of EMCCD.

While in the case of the high concentration of Ru(bpy)_3_^2+^ (500 μM), the ECL patterns generated at gold-coated UMEA change from ring to spot upon increasing the exposure time (see Supplementary Figure S5), which can be ascribed to the so-called “catalytic route” (Guo et al., 2020). In this case, TPrA^+•^ can be produced from the homogeneous chemical oxidation between TPrA and electrochemically generated Ru(bpy)_3_^3+^. In other words, the ECL patterns indeed depend on the effective diffusion distance of Ru(bpy)_3_^3+^.

### Theoretical Simulation

**FIGURE 6:**
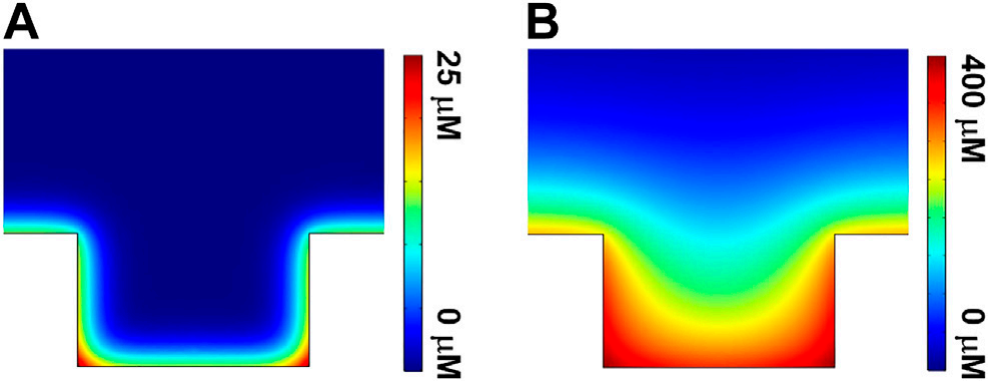
Side-view of simulated concentration contours of Ru(bpy)_3_^2+^* at a single microwell for the cases of **(A)** 50 μM and **(B)** 500 μM Ru(bpy)_3_^2+^.

To further rationalize the variation of ECL images obtained with gold-coated UMEA, the theoretical simulation using COMSOL software was employed to decipher the ECL reaction mechanisms (Sentic et al., 2014; Imai et al., 2015; Danis et al., 2018; Ma et al., 2018) at the single microwell (see more simulation details in Supplementary Material). Figure 6 shows the side-view of simulated concentration contours of Ru(bpy)_3_^2+*^ at a single microwell for low (**A**) and high (**B**) luminophore concentrations. In both cases, the maximum concentration of Ru(bpy)_3_^2+*^ was confined in the vicinity of the junction where the superposition of the diffusion fields exists (displayed as red color), because both bottom and sidewall of the microwell are coated by gold. The overlapped diffusion fields in radial and longitudinal directions favor the ECL reactions for light generation, thus enhancing the ECL intensities in the confined geometries. In addition, the distribution of excited state Ru(bpy)_3_^2+*^ at the bottom is denser than that at the top surface of UMEA. These simulation data are well consistent with experimental results.

## Conclusion

In summary, we have realized the visualization of the ECL reactions involving Ru(bpy)_3_^2+^ and TPrA at the single microwell level by combined use of the gold-coated UMEA and ECL imaging, spatially resolving the ECL generation of each microwell in the same solution. In both cases of low and high concentrations of Ru(bpy)_3_^2+^, the strongest emission is distributed at the junction between the sidewall and bottom of UMEA. Moreover, at the low concentration of Ru(bpy)_3_^2+^, the ECL pattern remains to be ring-shaped and is independent on the exposure time of EMCCD, because the shape of ECL image obtained on the microwells is determined by the limited lifetime of TPrA^+•^. In combination with theoretic simulation, the enhanced ECL generation in the confined microwells are rationalized to originate from the superposition of diffusion fields in both radial and longitudinal directions. This work provides a new perspective for studying the ECL reaction mechanisms with spatial resolution, and it can be employed for deciphering the ECL process involving other coreactants and promoting the detection sensitivity of immunomagnetic beads-based ECL assays with high-throughput analysis.

## Data Availability Statement

The original contributions presented in the study are included in the article/Supplementary Material, further inquiries can be directed to the corresponding authors.

## Author Contributions

BS and WG conceived and designed the study. JD fabricated gold-coated UMEA and completed ECL imaging test under the guidance of BS and WG. PZ and JD performed the theoretical simulation. All authors contributed to manuscript preparation and revision and have read and approved the submitted version.

## Funding

This work was supported by the National Natural Science Foundation of China (21904115, 21874117), the Natural Science Foundation of Zhejiang Province (LZ18B050001) and the China Postdoctoral Science Foundation (2020T130577, 2019M662019).

## Conflict of Interest

The authors declare that the research was conducted in the absence of any commercial or financial relationships that could be construed as a potential conflict of interest.
