# Supplementary material for: Confined Electrochemiluminescence Generation at Ultra-High-Density Gold Microwell Electrodes
Source: Front Chem. 2021 Jan 26;8:630246. doi: 10.3389/fchem.2020.630246 (PMC7870482; doi:10.3389/fchem.2020.630246)
Supplement: Supplementary file 1 [file datasheet1.docx]

Supplementary Material

# S1. Electrochemical Characterization of Gold-Coated Ultra-high-density of Microwell Electrode Array (UMEA)

**Supplementary Figure 1** shows the cyclic voltammograms (CVs) of UMEA in phosphate buffer (PB, 0.1 M, pH 7.4 black line) and the onset potential at ca. 0.5 V is attributed to the oxidation of gold-coated UMEA. The oxidation current of TPrA at UMEA surface sightly increases (green line). The anodic current peak at 0.86 V is ascribed to the oxidation of Ru(bpy)_3_^2+^ (blue line). The oxidation current peak of TPrA and Ru(bpy)_3_^2+^ can be observed clearly (red line).

**
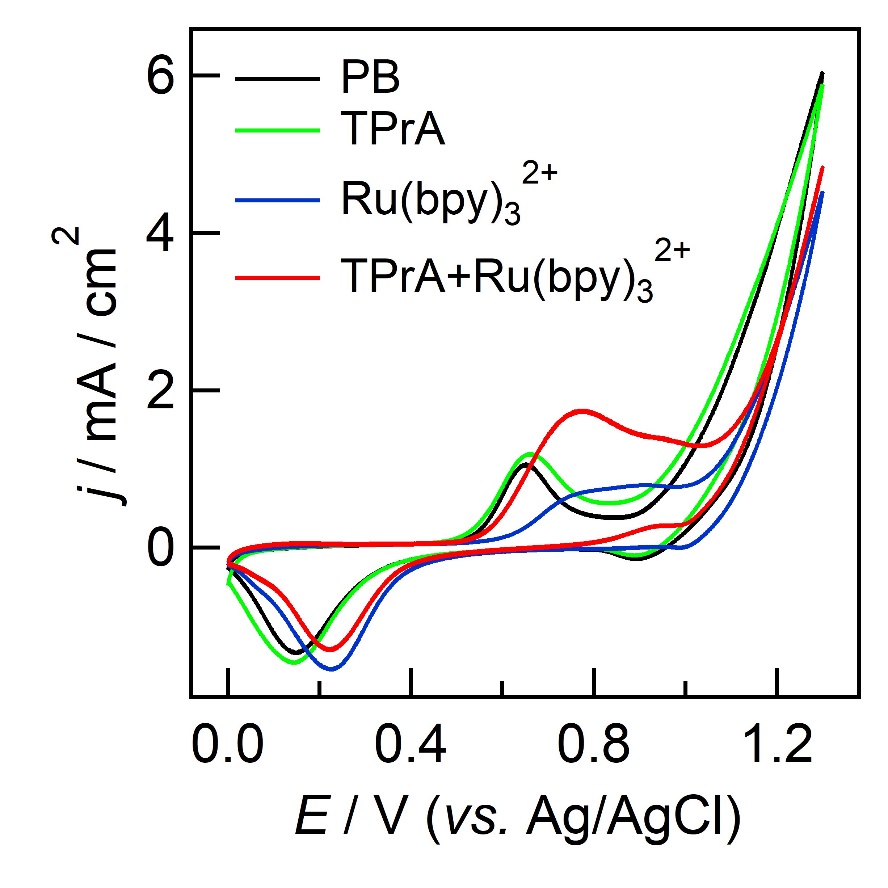
**

**Supplementary Figure 1.** CVs of gold-coated UMEA in phosphate buffer (PB, 0.1 M, pH 7.4) (black line), containing 25 mM TPrA (green line), 500 μM Ru(bpy)_3_^2+^ (blue line) and 25 mM TPrA/500 μM Ru(bpy)_3_^2+^ (red line). The scan rate was 0.1 V/s.

# S2. Stability of ECL Generation at Gold-Coated UMEA

**Supplementary Figure 2A-E** displays the ECL images of the same region of interest of UMEA obtained at different pulse time, and the variation of ECL intensity with time/cycle number is shown in **Figure 2F**. The relative standard deviation (RSD) of ECL intensity extracted from 10 single microwells is 6%, suggesting that the gold-coated UMEA possesses the good stability during the ECL measurement.


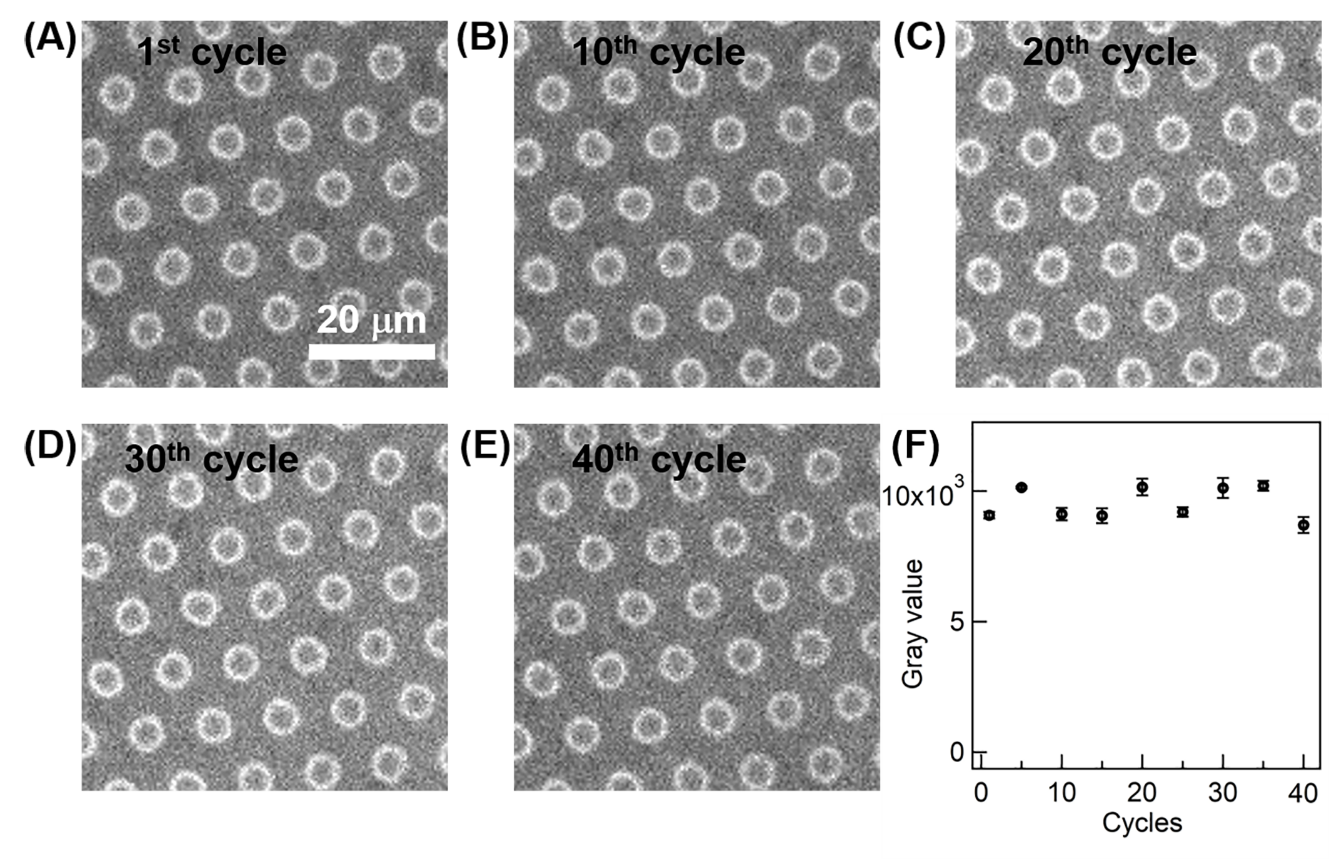


**Supplementary Figure 2.** (A-E) ECL images obtained with gold-coated UMEA in PB (0.1 M, pH 7.4) containing 50 μM Ru(bpy)_3_^2+^ and 50 mM TPrA obtained at different pulse time. (F) The variation of ECL intensity with time/cycle number obtained on the gold-coated UMEA. The ECL imaging was performed upon applying a double-step potential (initial potential 0 V; pulse potential 1.0 V; pulse period 2 s; pulse time 1 s). The exposure time of EMCCD was 4 s.

# S3. ECL Images Obtained with Gold-Coated UMEA and Gold Film Electrode

**Supplementary Figure 3** displays the ECL images obtained with gold-coated UMEA and commercial gold film electrode, respectively. Hundreds of ECL rings are homogenously distributed over the entire image (200 × 200 µm^2^). In addition, the ECL intensity from the former electrode is at least 7 times higher than the latter one.


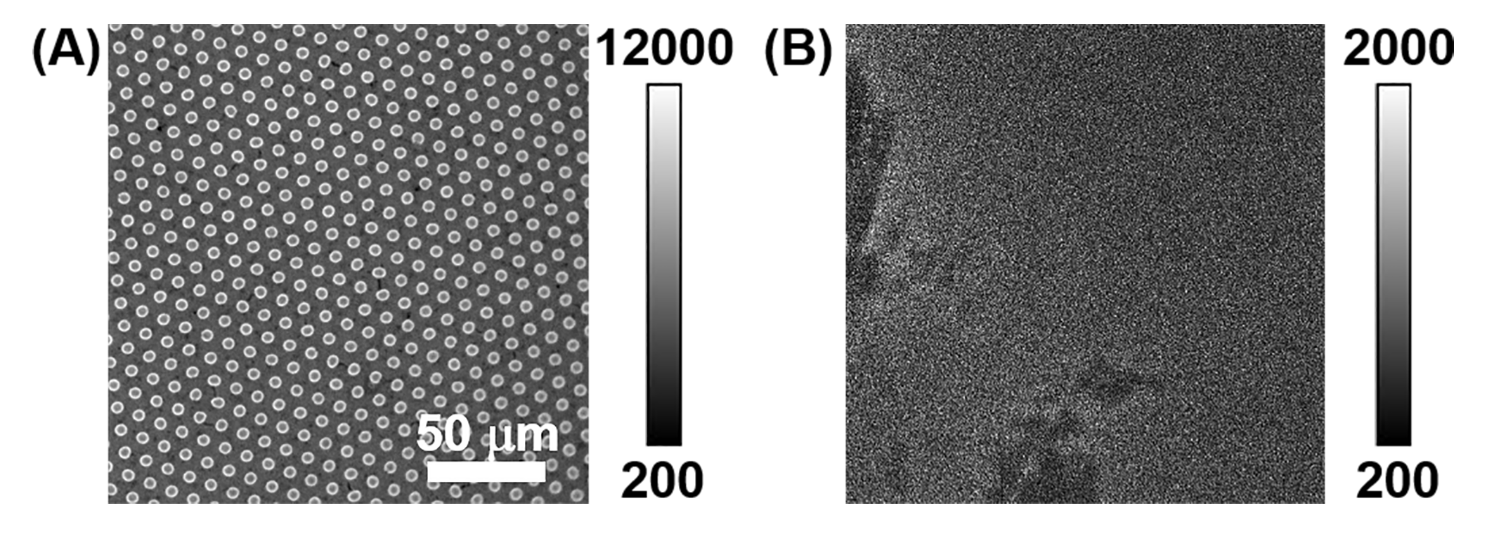


**Supplementary Figure 3.** ECL images obtained with the gold-coated UMEA (A) and commercial gold film electrode (B) in PB (0.1 M, pH 7.4) containing 50 μM Ru(bpy)_3_^2+^ and 50 mM TPrA. The ECL imaging was performed upon applying a double-step potential (initial potential 0 V; pulse potential 1.0 V; pulse period 2 s; pulse time 1 s). The exposure time of EMCCD was 4 s.

# S4. ECL Imaging of Gold-Coated UMEA at High Concentration of Ru(bpy)_3_^2+^

**Supplementary Figure 4A-F** compares BF and ECL images obtained at different pulse potentials for the same region of interest of UMEA. **Supplementary Figure 4G** displays the grayscale variations of ECL images along the radial direction of two adjacent microwells and **Supplementary Figure 4H** shows the variation of ECL intensity with the potential at the gold-coated UMEA at the single microwell level. The emission array shows the ring-shaped ECL patterns and the pulse potential of 1.1 V was optimized for ECL imaging.


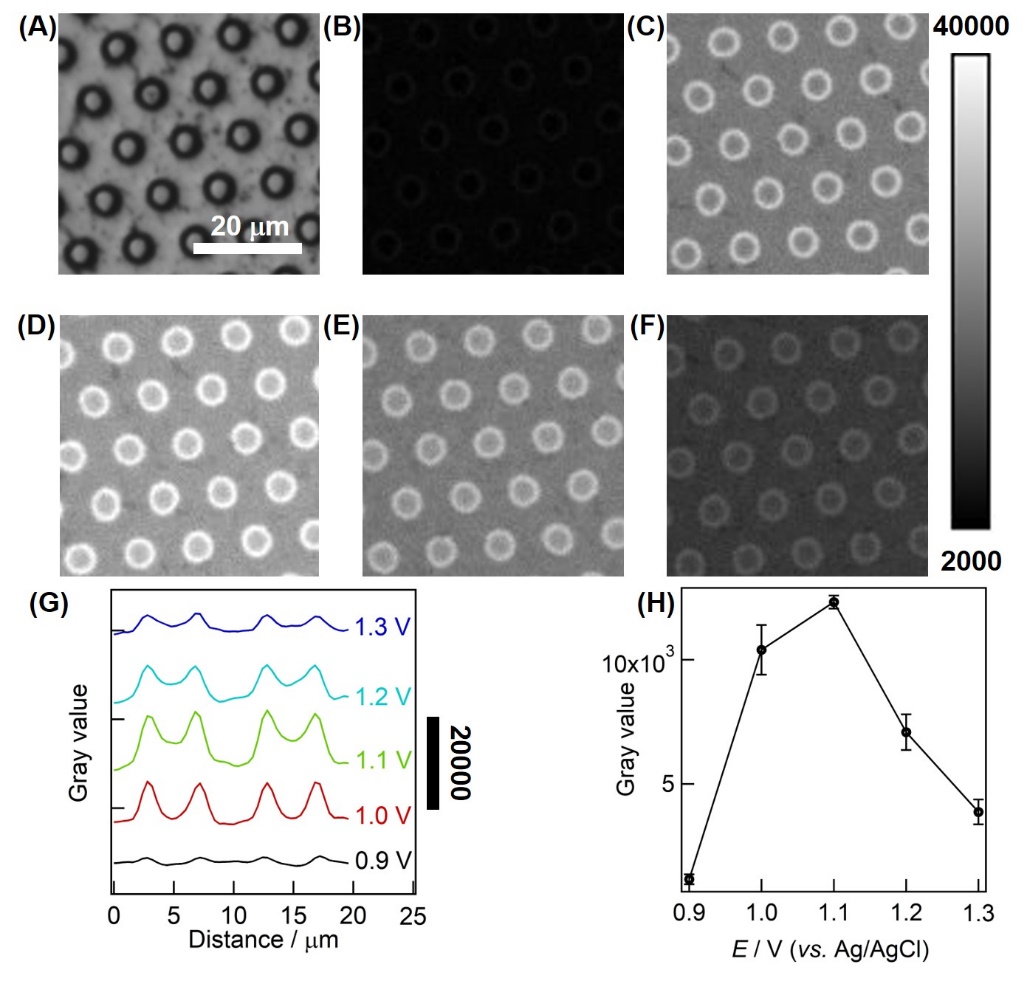


**Supplementary Figure 4.** Bright field (BF, A) and ECL (B-F) images of gold-coated UMEA in PB (0.1 M, pH 7.4) containing 500 μM Ru(bpy)_3_^2+^ and 25 mM TPrA. The ECL imaging was triggered by a double-step potential (initial potential 0 V; respective pulse potentials 0.9 V, 1.0 V, 1.1 V, 1.2 V, 1.3 V; pulse period 2 s; pulse time 1 s). The exposure time of EMCCD was 4 s. (G) The grayscale variation of ECL along the radial direction of two adjacent microwells. (H) The variation of ECL intensity with the potential on the gold-coated UMEA at the single microwell level.

# S5. Time-Evolution of ECL Imaging of Gold-Coated UMEA at the High Concentration of Ru(bpy)_3_^2+^

**Supplementary Figure 5A-F** displays the ECL images obtained with 500 µM Ru(bpy)_3_^2+^ and 25 mM TPrA under different exposure time, and the potential was pulsed to optimized value of 1.1 V. The ECL patterns generated at gold-coated UMEA changes from ring to spot upon increasing the exposure time, which can be ascribed to the so-called “catalytic route” (shown in **Supplementary Figure 5G)**. In this case, TPrA^+•^ can be produced from the homogeneous chemical oxidation reaction between TPrA and electrochemically generated Ru(bpy)_3_^3+^. As seen from **Supplementary Figure 5H**, the grayscale variation of ECL reach a plateau at the center of microwell with the increase of exposure time up to 4 s, suggesting the overlap of ECL emitting regions.


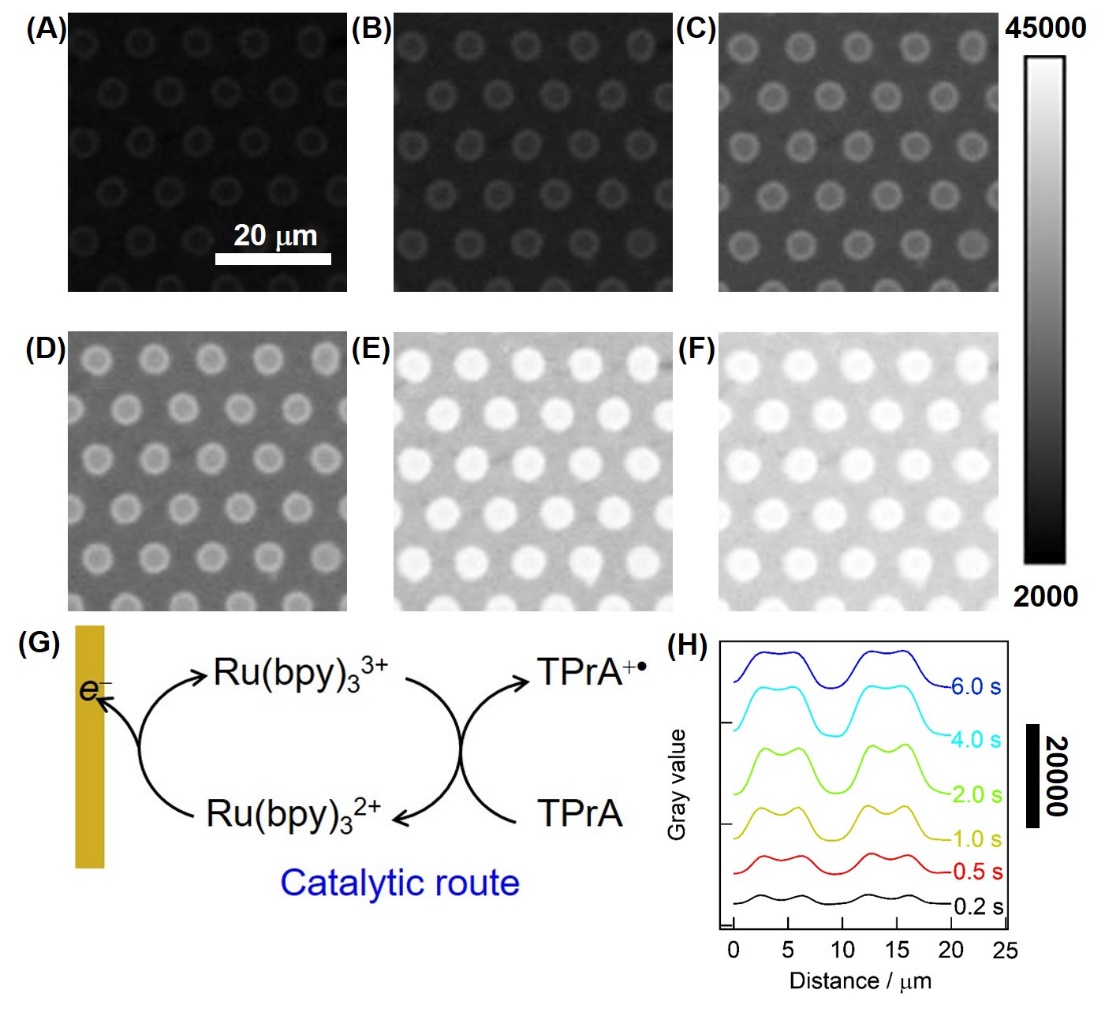


**Supplementary Figure 5.** (A-F) ECL images obtained with gold-coated UMEA in PB (0.1 M, pH 7.4) containing 500 μM Ru(bpy)_3_^2+^ and 25 mM TPrA. A double-step potential (initial potential 0 V, pulse potential 1.1 V) was applied to launch the ECL reactions. The pulse time was 0.2 s (A), 0.5 s (B), 1 s (C), 2 s (D), 4 s (E) and 6 s (F), respectively. The exposure time of EMCCD was in consistent with the period time of double-step potential. (G) The so-called “catalytic route” for ECL generation of Ru(bpy)_3_^2+^/TPrA system at a high luminophore concentration. (H) The grayscale variation of ECL along the radial direction of two adjacent microwells under different exposure time.

# S6. COMSOL Simulation

For simplifying the calculations in the simulation process, we only take the main routes involved into account. In case of low concentration of Ru(bpy)_3_^2+^, the direct oxidation of Ru(bpy)_3_^2+^ and TPrA is the main ECL path, because the kinetic rate of homogeneous reaction between Ru(bpy)_3_^3+^ and TPrA^•^ is larger than other reactions **(Danis et al., 2018)**. This ECL process includes equations 1-6 in **Supplementary Table 1**. For high concentration of Ru(bpy)_3_^2+^, the “catalytic route” is additionally considered for ECL generation, corresponding to equation 7.

**Supplementary Table 1** All reactions involving in the ECL process.

| Category | Reaction equation |  |
| --- | --- | --- |
| Charge transfer reactions | **** | (1) |
|  | **** | (2) |
|  | **** | (3) |
| Homogeneous reactions | **** | (4) |
|  | **** | (5) |
|  | **** | (6) |
|  | **** | (7) |

Theoretical simulations were carried out by the commercial finite element software COMSOL Multiphysics (Version 5.2). 2D axisymmetric model was built to simulate the physical geometry in ECL simulations, as shown in **Supplementary Figure 6A**. Because the radial diffusion from multiple micropores are not be overlapped, we performed digital simulations of a single microwell to simplify the simulation process. “Transport of Diluted Species” physical field is used to study the time-dependent transportation of involved species in the ECL reactions. As seen in **Supplementary Figure 6B**, a refined mesh was set on the electrode surface boundaries.


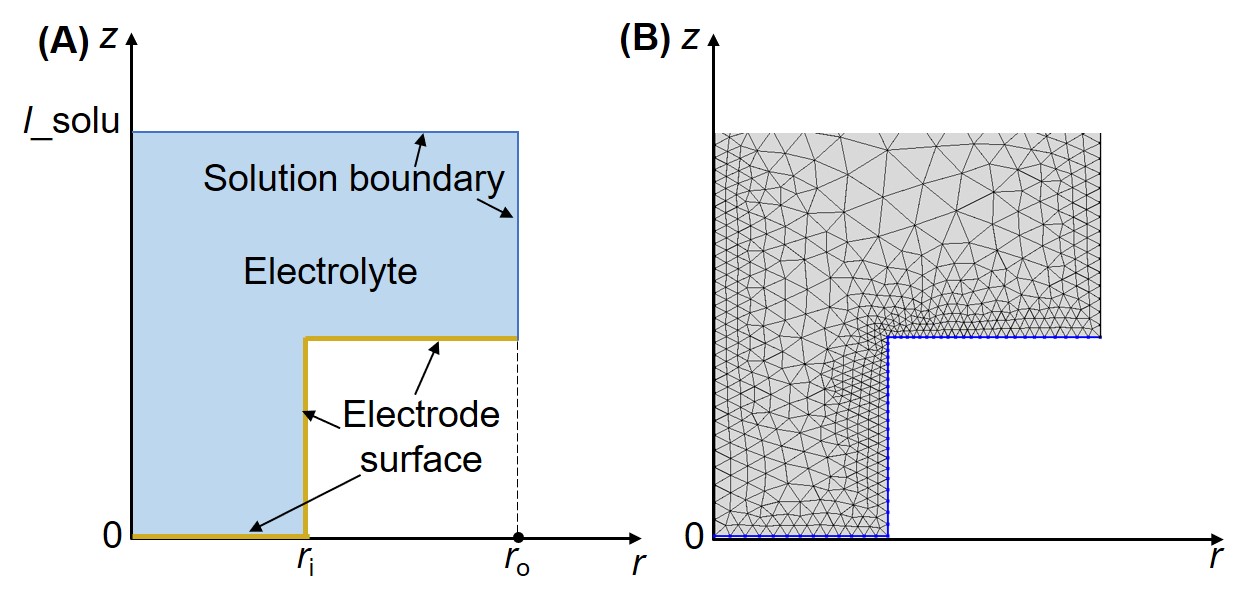


**Supplementary Figure 6.** (A) 2D axisymmetric model employed in the COMSOL simulation (not to real scale). *r* and *z* are the coordinates in the directions parallel and normal to the substrate surface, respectively. *r*_i_ is the inner radii of the single microwell. *r*_o_ is the width of physical geometry. (B) Mesh settings in COMSOL simulation. The blue line represents the conductive area.

All parameters used in simulation are summarized in **Supplementary Table 2**. The kinetic and thermodynamic parameters including diffusion coefficient and rate constant were referred to previously reported works **(Miao et al., 2002; Sentic et al., 2014; Imai et al., 2015; Ma et al., 2018; Guo et al., 2020)**.

**Supplementary Table 2** Simulation parameters in global definitions.

| Category | Name | Value | Description |
| --- | --- | --- | --- |
| Boundary conditions | *r*_o_ | 4.915 μm | The width of physical geometry |
|  | *r*_i_ | 2.175 μm | The radius of single micropore |
|  | *l*_ele | 2.5 μm | The height of single micropore |
|  | *l*_solu | 3 mm | The length of axial solution boundary |
| Initial concentration | *c*_0__Ru^2+^ | 500 μM / 50 μM | Initial concentration of Ru(bpy)_3_^2+^ |
|  | *c*_0__TPrA | 25 mM / 50 mM | Initial concentration of TPrA |
| Diffusion coefficient | *D*_Ru_ | 5.9◊10^−6^ cm^2^/s | Diffusion coefficient of Ru(bpy)_3_^2+^, Ru(bpy)_3_^3+^, Ru(bpy)_3_^2+∗^ |
|  | *D*_TPrA_ | 5.0◊10^−6^ cm^2^/s | Diffusion coefficient of TPrA, TPrA^+•^, TPrA^•^ |
| Reaction rate | *k*_1_ | 0.06 cm^2^/s | Rate constant of eq (1) |
|  | *k*_2_ | 0.01 cm^2^/s | Rate constant of eq (2) |
|  | *k*_3_ | 0.01 cm^2^/s | Rate constant of eq (3) |
|  | *k*_4_ | 3500 s^−1^ | Rate constant of eq (4) |
|  | *k*_5_ | 1.0◊10^9^ (M·s)^−1^ | Rate constant of eq (5) |
|  | *k*_6_ | 300 s^−1^ | Rate constant of eq (6) |
|  | *k*_7_ | 1.3◊10^4^ (M·s)^−1^ | Rate constant of eq (7) |

For the reaction that occurs on the electrode surface, they are sufficiently fast so that the oxidations proceed under the diffusion control. Thus, the change in substance concentration at the surface of electrode can be described as equations 8-10 in **Supplementary Tables 3 and 4**. In the solution conditions, molecules transport exclusively by diffusion. Thus, the transition conditions for all redox species can be expressed by the Fick’s second law. According to the different paths at low and high concentrations of Ru(bpy)_3_^2+^, the concentration change of each substance in the solution can be expressed as eqs. 11-15 and 16-21 in **Supplementary Tables 3 and 4**, respectively.

**Supplementary Table 3** The concentration change of all substances under the low concentration of Ru(bpy)_3_^2+^.

| Category | Expression |  |
| --- | --- | --- |
| Electrode surface | **$D_{\text{Ru}}\frac{\partial\text{[Ru}^{\text{2+}}\text{]}}{\partial\text{r}}=-D_{\text{Ru}}\frac{\partial\text{[Ru}^{\text{3+}}\text{]}}{\partial\text{r}}=-k_{1}[\text{Ru}^{\text{2+}}]$** | (8) |
|  | **$D_{\text{TPrA}}\frac{\partial\text{[TPrA]}}{\partial\text{r}}=-D_{\text{TPrA}}\frac{\partial\text{[TPrA}^{\text{+}\bullet}\text{]}}{\partial\text{r}}=-k_{2}[\text{TPrA}]$** | (9) |
|  | **$D_{\text{TPrA}}\frac{\partial\text{[TPrA}^{\bullet}\text{]}}{\partial\text{r}}=-D_{\text{TPrA}}\frac{\partial\text{[P}_{0}\text{]}}{\partial\text{r}}=-k_{3}[\text{TPrA}^{\bullet}]$** | (10) |
| Solution | **** | (11) |
|  | **** | (12) |
|  | **** | (13) |
|  | **** | (14) |
|  | **** | (15) |

**Supplementary Table 4** The concentration change of all substances under the high concentration of Ru(bpy)_3_^2+^.

| Category | Expression |  |
| --- | --- | --- |
| Electrode surface | **$D_{\text{Ru}}\frac{\partial\text{[Ru}^{\text{2+}}\text{]}}{\partial\text{r}}=-D_{\text{Ru}}\frac{\partial\text{[Ru}^{\text{3+}}\text{]}}{\partial\text{r}}=-k_{1}[\text{Ru}^{\text{2+}}]$** | (8) |
|  | **$D_{\text{TPrA}}\frac{\partial\text{[TPrA]}}{\partial\text{r}}=-D_{\text{TPrA}}\frac{\partial\text{[TPrA}^{\text{+}\bullet}\text{]}}{\partial\text{r}}=-k_{2}[\text{TPrA}]$** | (9) |
|  | **$D_{\text{TPrA}}\frac{\partial\text{[TPrA}^{\bullet}\text{]}}{\partial\text{r}}=-D_{\text{TPrA}}\frac{\partial\text{[P}_{0}\text{]}}{\partial\text{r}}=-k_{3}[\text{TPrA}^{\bullet}]$** | (10) |
| Solution | **** | (16) |
|  | **** | (17) |
|  | **** | (18) |
|  | **** | (19) |
|  | **** | (20) |
|  | **** | (21) |

[Ru^2+^], [Ru^3+^], [Ru^2+*^], [TPrA], [TPrA^•^] and [TPrA^+•^] are the concentration of Ru(bpy)_3_^2+^, Ru(bpy)_3_^3+^, Ru(bpy)_3_^2+*^, TPrA, TPrA^•^ and TPrA^+•^, respectively. Δ is the Laplacian in the 2D axisymmetric geometry.

# References

Danis, A.S., Potts, K.P., Perry, S.C., and Mauzeroll, J. (2018). Combined spectroelectrochemical and simulated insights into the electrogenerated chemiluminescence coreactant mechanism. *Anal. Chem.* 90, 7377−7382. doi: 10.1021/acs.analchem.8b00773

Guo, W.L., Zhou, P., Sun, L., Ding, H., and Su, B. (2020). Microtube electrodes for imaging the electrochemiluminescence layer and deciphering reaction mechanism. *Angew. Chem. Int. Ed.* in press, doi: 10.1002/anie.202012340

Imai, K., Valenti, G., Villani, E., Rapino, S., Rampazzo, E., and Marcaccio, M. (2015). Numerical simulation of doped silica nanoparticle electrochemiluminescence. *J. Phys. Chem. C* 119, 26111−26118. doi: 10.1021/acs.jpcc.5b07107

Ma, C., Wu, W.W., Li, L.L., Wu, S.J., Zhang, J.R., and Chen, Z.X. (2018). Dynamically imaging collision electrochemistry of single electrochemiluminescence nano-emitters. *Chem. Sci.* 9, 6167−6175. doi: 10.1039/c8sc02251h

Miao, W.J., Choi, I.P., and Bard, A.J. (2002). Electrogenerated chemiluminescence 69: the tris(2,2′-bipyridine)ruthenium(II), (Ru(bpy)_3_^2+^)/tri-n-propylamine (TPrA) system revisited−a new route involving TPrA^•+^ cation radicals. *J. Am. Chem. Soc.* 124, 14478−14485. doi: 10.1021/ja027532v

Sentic, M., Milutinovic, M., Kanoufi, K., Manojlovic, D., Arbaulta, S., and Sojic, N. (2014). Mapping electrogenerated chemiluminescence reactivity in space: mechanistic insight into model systems used in immunoassays. *Chem. Sci.* 5, 2568−2572. doi: 10.1039/c4sc00312h
